# Supplementary material for: Do Metabolically Healthy People with Obesity Have a Lower Health-Related Quality of Life? A Prospective Cohort Study in Taiwan
Source: J Clin Med. 2021 Oct 30;10(21):5117. doi: 10.3390/jcm10215117 (PMC8584400; doi:10.3390/jcm10215117)
Supplement: Supplementary file 1 [file jcm-10-05117-s001.zip › jcm-1387209-supplementary.pdf]

Table S1. Demographic characteristics at follow-up of 427 participants with complete follow-up assessment, classified by the metabolic health/BMI groups at follow-up.

	Total	Metabolically healthy normal weight [a]	Metabolically healthy overweight [a]	Metabolically healthy obesity [a]	Metabolically un-healthy normal weight	Metabolically un-healthy overweight	Metabolically un-healthy obesity	*p*
	n = 427	*n* = 195 (45.7%)	*n* = 52 (12.2%)	*n* = 21 (4.9%)	*n* = 55 (12.9%)	*n* = 45 (10.5%)	*n* = 59 (13.8%)	
Age at follow-up, years, *n* (%)								
43–49	80 (19.2)	37 (19.0)	12 (23.1)	6 (28.6)	4 (7.3)	6 (13.3)	17 (28.8)	
50–59	221 (51.6)	106 (54.4)	28 (53.9)	12 (57.1)	26 (47.3)	25 (55.6)	24 (40.7)	0.04
60–65	125 (29.2)	52 (26.7)	12 (23.1)	3 (14.3)	25 (45.5)	14 (31.1)	18 (30.5)	
Mean age (SD)	55.8 (5.4)	55.5 (5.4)	55.6 (5.2)	54.4 (5.4)	58.0 (4.7)	56.3 (5.2)	55.4 (5.9)	
Women, *n* (%)	253 (59.3)	147 (75.4)	21 (41.2)	9 (42.9)	34 (61.8)	21 (46.7)	20 (33.9)	<0.001
Mean BMI (SD), kg/m^2	23.9 (4.0)	21.0 (1.7)	25.2 (0.8)	29.3 (2.3)	22.4 (1.3)	25.5 (0.9)	30.6 (3.8)	
Married, *n* (%)	366 (86.3)	162 (83.5)	46 (90.2)	19 (90.5)	48 (88.9)	39 (88.6)	51 (86.4)	0.75
Alcohol consumption, *n* (%)	86 (20.2)	28 (14.5)	12 (23.1)	6 (28.6)	16 (29.1)	9 (20.0)	15 (25.4)	
Cigarette smoking, *n* (%)								
Non-smokers	361 (84.7)	174 (89.7)	44 (84.6)	18 (85.7)	47 (85.5)	35 (77.8)	43 (72.9)	
Smokers	35 (8.2)	12 (6.2)	5 (9.6)	1 (4.8)	6 (10.9)	5 (11.1)	6 (10.2)	0.07
Ex-smokers	30 (7.0)	8 (4.1)	3 (5.8)	2 (9.5)	2 (3.6)	5 (11.1)	10 (17.0)	
Physical activity [b], *n* (%)								
Low	221 (51.6)	89 (45.6)	32 (61.5)	13 (61.9)	26 (47.3)	25 (55.6)	35 (59.3)	
Moderate	149 (34.8)	74 (38.0)	13 (25.0)	5 (23.8)	21 (38.2)	16 (35.6)	20 (33.9)	0.41
High	58 (13.6)	32 (16.4)	7 (13.5)	3 (14.3)	8 (14.6)	4 (8.9)	4 (6.8)	
Education, *n* (%)								
Illiterate/elementary school	9 (2.1)	4 (2.1)	1 (2.0)	0	1 (1.8)	0	3 (5.1)	
Senior/junior high school	153 (35.9)	54 (27.7)	22 (43.1)	8 (38.1)	25 (45.5)	22 (48.9)	22 (37.3)	0.09
University and above	264 (62.0)	137 (70.3)	28 (54.9)	13 (61.9)	29 (52.7)	23 (51.1)	34 (57.6)	
SF-36 (mean ± SD)								
PCS	52.9 (6.2)	53.1 (6.1)	53.5 (6.0)	53.7 (6.4)	53.1 (5.9)	52.9 (5.5)	51.1 (7.0)	0.34

MCS	48.7 (8.2)	49.0 (7.9)	48.5 (7.9)	48.0 (10.1)	48.1 (8.2)	49.3 (8.0)	47.8 (9.2)	0.90
Physical functioning	53.0 (5.0)	53.1 (4.9)	53.8 (4.6)	52.5 (5.6)	54.0 (4.0)	52.7 (4.9)	51.6 (6.0)	0.11
Role-physical	51.7 (8.6)	52.5 (8.1)	51.2 (8.6)	50.5 (10.2)	50.6 (8.9)	53.3 (7.2)	49.9 (10.2)	0.21
Bodily pain	53.3 (8.0)	52.9 (7.7)	53.8 (7.8)	56.4 (7.7)	53.4 (8.4)	53.2 (8.4)	53.2 (8.9)	0.59
General health	47.1 (8.3)	47.7 (8.3)	47.7 (7.1)	46.9 (9.1)	47.1 (8.9)	47.9 (8.8)	43.9 (7.9)	0.07
Vitality	54.0 (7.5)	54.1 (7.1)	54.4 (7.3)	53.1 (8.9)	54.3 (8.2)	54.4 (7.5)	52.9 (7.9)	0.87
Social functioning	50.3 (6.1)	50.8 (6.1)	50.0 (6.4)	48.9 (6.3)	50.4 (5.5)	50.0 (6.2)	49.5 (6.6)	0.64
Role-emotional	50.6 (9.5)	51.2 (8.9)	51.0 (9.0)	48.8 (11.3)	50.0 (9.9)	52.0 (8.1)	48.8 (11.5)	0.44
Mental health	47.0 (8.2)	46.9 (8.1)	46.4 (7.3)	49.1 (8.5)	46.9 (8.5)	47.7 (8.2)	46.7 (9.0)	0.83

Note. SD=standard deviation; BMI= body mass index; SF-36 = the Short Form Health Survey; PCS=physical component summary; MCS=mental component summary. Chi-square tests and Fisher's exact tests were used for categorical variables. Unbalanced ANOVA tests were used for continuous variables. [a] Metabolic health was defined as (1) absence of known chronic diseases including hypertension, hyperlipidemia, diabetes, coronary artery disease, stroke and vascular diseases; (2) presence of ≤ 1 metabolic risk factor, including hypertension, hyperglycemia, hypertriglyceridemia, and low serum high-density lipoprotein cholesterol. [b] Physical activity was evaluated by the International Physical Activity Questionnaire (IPAQ) Short-Form, Taiwan version.

Table S2. The association between metabolic health/BMI groups and HRQOL with different reference groups.

Model A	Metabolically healthy overweight	Metabolically unhealthy overweight
PCS	Reference group	0.37 (−0.96, 1.70)
MCS	Reference group	0.64 (−0.89, 2.18)
Physical functioning	Reference group	0.10 (−0.87, 1.07)
Role-physical	Reference group	2.34 (0.57, 4.12)
Bodily pain	Reference group	0.51 (−1.32, 2.35)
General health	Reference group	-0.42 (−2.31, 1.48)
Vitality	Reference group	-0.34 (−1.59, 0.90)
Social functioning	Reference group	0.21(−1.59, 2.01)
Role-emotional	Reference group	1.51(−0.51, 3.54)
Mental health	Reference group	0.83(−0.64, 2.31)
**Model B**	**Metabolically healthy obesity**	**Metabolically unhealthy obesity**
PCS	Reference group	-3.37 (-5.16, -1.59)
MCS	Reference group	1.53 (-0.28, 3.33)
Physical functioning	Reference group	-0.47 (-1.88, 0.94)
Role-physical	Reference group	-2.25 (-4.55, 0.04)
Bodily pain	Reference group	-2.87 (-5.15, -0.59)
General health	Reference group	-4.94 (-7.25, -2.63)
Vitality	Reference group	1.27 (-0.36, 2.89)
Social functioning	Reference group	1.77 (-0.33, 3.86)
Role-emotional	Reference group	-1.26 (-3.83, 1.32)
Mental health	Reference group	0.94 (-0.84, 2.72)

Note. BMI = body mass index; HRQOL= health-related quality of life; PCS= physical component summary; MCS= mental component summary. The reference group was metabolically healthy overweight in model A and metabolically healthy obesity in model B. The analyses were conducted by generalized linear mixed models with adjustment for age, sex, marital status, level of education, smoking, alcohol consumption, groups of physical activity, and follow-up years.
